# Foreign Correspondence

**Published:** 1890-01

**Authors:** 


### Foreign Correspondence.

To the Editor:

Though this Hawaiian kingdom is but a dot in the great Pacific Ocean, there are gathered into it perhaps a greater variety of nationalities than exist in any other land. From the Swede of the North to the inhabitant of the Atolls of the South Seas—from the Indians of the East to the dwellers in the land of Sinim, all gather upon these sunny shores, and come, more or less, under our observation and care. By the near roar of the breakers upon our encircling reefs, we are thrown into close relations and learn much of each other's ways and character. It has occurred to me that some observations, made during my twenty years' practice among this people, might not be without interest to your readers.

The Hawaiians of the past had good teeth, but by no means the perfect teeth that writers have so often told us were the especial boon of all savage races. Fortunately for the student in such matters, the ancient Hawaiians were often buried in the almost inaccessible lava caves with which some parts of the country abound, or in the shifting sands of the sea-shore, which, sooner or later, leave the whitened bones exposed to the observer. In the hundreds of crania which I have examined, in which the superior and inferior maxillaries were together, I have found few cases where there were not greater or less effects of dental caries, not infrequently salivary calculi, and, in a few instances, unmistakable evidences of pyorrhœa alveolaris. In one cranium in my possession there were eight alveolar abscesses in the superior maxillary at the time of death. Though, on the whole, the teeth of these ancient people, as well as those of the present generation, are very regular, the inferior third molar seems to have been a frequent cause of great misery. I have one specimen in which this crowded *dens sapientiæ* has retaliated by causing complete anchylosis of the jaw. The second molar was forced out, evidently to give space for the individual to be fed.

The Hawaiians in middle life to-day have better teeth than their European neighbors; but their children have very little advantage over their white playmates. I attribute this rapid deterioration to the fact that while their parents ate much the same food that their fathers used before civilization came to them, the children, using the less simple foreign diet, with little personal attention to their teeth, are more subject to dental diseases.

The Chinese (large numbers of whom, of the better classes, have been under my care) are quite similar to the Hawaiians in the soundness and strength of their teeth, but from a different cause. These people, evidently, from ages, have taken great care of their teeth, as far as personal attention could go. The majority of those whom I have met who would be called the upper class are afflicted with *pyorrhœa alveolaris*, while the laborers are no more subject to it than other nationalities. I attribute this in a great measure to the free use of their tooth-powder, which consists of finely powdered pumice or silicon. This in time separates the gum from the teeth, and paves the way for more serious trouble. Irregularities, especially in the third molars, are common among all classes.

I have examined with great interest the teeth of the South Sea Islanders, especially those coming from the low islands, where little but cocoanuts and bread-fruit will grow. So far as my observation extends, they have large teeth, but of very poor quality. The teeth have a yellow, opaque look, with soft enamel, which readily wears down and breaks off. The dentine has that peculiar tallowy cut well known to the dentist as not hopeful for long retaining the filling. The secretions of the mouth seem normal, so that these teeth, indifferent as they are, remain to do good service, giving them little trouble, except from periostitis. The teeth of these people have not been influenced by civilization.

We often hear the teeth of the American people represented as more frail than those of their cousins across the Atlantic. But it is my observation from a cosmopolitan practice that they are not inferior to any, and are superior to several of the European nationalities. One reason why this sentiment has prevailed among travellers may be that Americans take more care of their teeth, and show their care both by their conversation and the evident appearance of the mouth.

J. M. Whitney.

Honolulu.

---

To the Editor:

The letter of Dr. Darley, in the October number of the International Dental Journal, induces me to give the conclusions I have arrived at from an experimental use of Watt's crystal gold. When I say an experimental use, I mean that I have used up about four packages of it, that fillings of it were packed in various ways, and that a considerable number were made out of the mouth in cavities cut in bone or ivory. The fillings that I made out of the mouth were treated for cohesion and toughness by cutting with excavators, for surface hardness by firm burnishing, and for adaptation by removing the filling intact and examining it with a magnifying glass. I found that it was possible to arrive at perfect adaptation if very small pieces were used and very great care was taken: a better adaptation, in fact, than I was able to obtain by the ordinary methods of working foil. Burnishing pellets of either cohesive or non-cohesive foil being the only means by which I could get as good results. The only apparent difference being that the "burnished-to-place" gold was bright and polished where it was in contact with the cavity walls, and the crystal gold was quite dull. To obtain this good adaptation with crystal gold requires more time and care than the average practitioner can give. Unless this minute care is taken, and minute pieces used, it is, I believe, inferior in this respect to foil. The hardness of the filling is greater than those ordinarily made of foil, but the cohesion and toughness of the filling is decidedly inferior. To prove this, make two fillings, one of crystal gold and one of foil. Use relatively small pieces of each. Then try to dig out the gold with a moderately strong excavator. Don't just pick at the surface, but press the sharp blade in with force at an angle of forty-five degrees and try to lever up the layers of gold. The difference is at once apparent. These experiments and others lead me to the conclusion that it is an easy material with which to make inferior fillings in a short time; but that, in order to do superior work, it necessitates more care and time than foil; and that, no matter what care is taken, the filling is not so absolutely cohesive as if foil were used. The cohesion may, however, be sufficient for all practical purposes. The instruments I used were very finely serrated.

WM. C. GRAYSTON, L.D.S.

SCARBOROUGH, ENGLAND.

